# Clinical characteristics and etiology of children with bronchiolitis before and during the COVID-19 pandemic in Suzhou, China

**DOI:** 10.3389/fped.2022.974769

**Published:** 2022-11-14

**Authors:** Xiaohui Jiang, Ting Wang, Ge Dai, Huiming Sun, Wujun Jiang, Zhengrong Chen, Yongdong Yan

**Affiliations:** ^1^ Department of Respiratory Medicine, Children’s Hospital of Soochow University, Suzhou, China; ^2^ Department of Pediatrics, Huaian Hospital of Huaian City, Huaian, China

**Keywords:** bronchiolitis, clinical characteristics, etiology, COVID-19 pandemic, respiratory syncytial virus

## Abstract

**Objective:**

We sought to compare the clinical characteristics and etiology of children with bronchiolitis in Suzhou before the pandemic of coronavirus disease 2019 (COVID-19) with those during the pandemic.

**Methods:**

Children who were hospitalized with bronchiolitis in the Department of Respiratory Disease, Children's Hospital of Soochow University were retrospectively enrolled over 3 consecutive years (2019, 2020, and 2021) from February 1 to January 31. Medical records were reviewed for etiology, clinical manifestations, and laboratory examination results.

**Results:**

The pathogen detection rate and the positive respiratory syncytial virus (RSV) detection rate were lowest in 2020 and highest in 2021. The rate of human rhinovirus detection in 2021 was higher than that in 2019 but similar to that in 2020. The RSV-positive rate differences among the 3 years varied by age group. Regarding the monthly distribution of RSV-positive cases over the 3-year study, all age groups showed a significant increase in the number of cases during the winter of 2021, and this increase started as early as October. With regard to clinical manifestations, the proportion of children presenting with stuffy nose rhinorrhea in 2021 [73.33% (165/225)] was greater than that in 2019 [48.61% (122/251)] and 2020 [57.06% (97/170)], while the proportion of children with gastrointestinal symptoms in 2021 [11.56% (26/225)] was smaller than that in 2019 [25.50% (64/251)] but similar to that in 2020 [17.06% (29/170)].

**Conclusions:**

After the implementation of COVID-19 pandemic-related interventions, significantly lower pathogen detection and RSV-positive rates were observed in children with bronchiolitis in 2020. An upward trend in these rates was observed in 2021, coinciding with the relaxation of COVID-19 prevention measures. Strengthening infection control and surveillance systems is extremely important for future work.

## Introduction

Bronchiolitis, an acute infection of the lower respiratory tract involving mainly the bronchioles, is one of the most substantial disease burdens for infants and young children worldwide (1). It is a common clinical syndrome occurs in children under 2 years of age, most often in the first year of life. A typical disease course begins with rhinitis and cough, which may progress to tachypnea, wheezing, rales, use of accessory muscles, grunting, or nasal flaring (2). Viral infection is the main cause of bronchiolitis; the most common causative viruses are respiratory syncytial virus (RSV), followed by human rhinovirus (HRV), parainfluenza virus (PIV, especially PIV III), adenovirus (ADV), influenza virus, human metapneumovirus (hMPV), and human bocavirus (HBoV) (3). Additionally, *Mycoplasma pneumoniae* (MP), *Chlamydia pneumoniae* (CP) and *Bordetella pertussis* (B. pertussis) can also cause bronchiolitis (4–7).

At the end of 2019, a novel coronavirus, severe acute respiratory syndrome coronavirus 2 (SARS-CoV-2) was identified in hospitalized patients in Wuhan, China, and the illness caused by this virus was later named coronavirus disease 2019 (COVID-19) (8). In late January 2020, Suzhou municipal government implemented the first-level public health emergency response and ordered a stringent lockdown. Measures, such as prohibiting people from gathering, closing public places, postponing the resumption of work and school, wearing masks, and maintaining physical distancing, were taken to prevent the rapid spread of SARS-CoV-2. These measures impacted not only COVID-19, but also the dynamics of various other infectious diseases, including bronchiolitis (9, 10). However, it is unclear whether, in the second year of the pandemic when the prevention measures were relaxed, the pathogens associated with bronchiolitis continued to be markedly reduced and whether the clinical manifestations changed.

Because Suzhou municipal government enacted lockdown measures at the end of January 2020, here, we took January 31, 2020 as the time node and compared the clinical characteristics and etiology of children hospitalized with bronchiolitis over 3 consecutive years, i.e., 2019 (pre-pandemic), 2020 (first year of the pandemic), and 2021 (second year of the pandemic), from February 1 to January 31.

## Research subjects and methods

### Subjects

We conducted a retrospective analysis of the data from children who were admitted to the Department of Respiratory Disease at the Children's Hospital of Soochow University with a final diagnosis of bronchiolitis during the period from February 1 to January 31 in 2019, 2020, and 2021. It is important to note that only patients with negative SARS-CoV-2 nucleic acid tests could be admitted to the hospital. The inclusion criteria for this study were: age of between 1 month and 1 year old; occurrence of first episode of wheezing; and clinical evidence of bronchiolitis (tachypnea, wheeze, prolonged expiratory phase, and crackles on auscultation). The exclusion criteria were: recurrent wheezing; bronchopulmonary dysplasia; neuromuscular disease; congenital disease; congenital immunodeficiency; or evidence suggesting that the wheezing is caused by non-infectious factors, such as a bronchial foreign body. This study was approved by the Medical Ethics Committee of the Children's Hospital of Soochow University.

### Specimen collection

Nasopharyngeal secretions, collected from all children within 24 h of hospital admission, were analyzed. For aspiration, a suction catheter was introduced through the nose and advanced into the lower portion of the pharynx, up to a distance of approximately 7 cm–9 cm, and approximately 2 ml of nasopharyngeal secretions were obtained and sent for analysis within 30 min. The retrieved specimen was centrifuged (500 g, 10 min) and suspended in 2 ml saline before being divided into two aliquots for pathogen identification using a direct immunofluorescence assay (DFA) and polymerase chain reaction (PCR).

### Microbe detection

Seven common respiratory viruses, i.e., RSV, influenza virus A, influenza virus B, PIV I, PIV II, PIV III, and ADV were all detected using DFA. Chemicon (United States) provided the assay kits, and all staining methods were carried out according to the manufacturer's instructions. After that, immunofluorescence experiments were carried out (Leica 020-518.500, Germany). Trizol reagent (Invitrogen, United States) was used to extract RNA from the specimens, which was followed by reverse transcription to create cDNA. The cyclic temperature settings were 94 °C for 30 s, 55 °C for 30 s, 68 °C for 30 s, and a final extension at 68 °C for 7 min after 45 amplification cycles. A fluorescent real-time PCR (BIO-RAD iCycler) was used to detect hMPV and HRV. To identify HBoV, DNA extraction and real-time PCR were used. The 16 s rRNA gene of MP extracted from nasopharyngeal samples was identified using a quantitative diagnostic kit (Daan Gene Co., Ltd. Of Sun Yat-Sen University) for MP DNA.

### Data collection

The medical records of the patients were reviewed, and data regarding the following parameters were recorded: (1) demographic and clinical characteristics, including age, gender, symptom duration prior to admission, and hospital stay length; (2) results of blood tests for inflammatory profiles, including white blood cell (WBC) count, neutrophil percentage, and serum C-reactive protein (CRP) level; and (3) results of etiological examination of nasopharyngeal secretions. Tachypnea was defined as follows: >60 breaths/min in children aged <2 months, >50 breaths/min in children aged 2–11 months.

### Statistical analysis

Statistical analyses were performed using the Statistical Package for the Social Science (version 26.0, IBM Corporation, Armonk, NY, United States). Descriptive continuous outcome variables are presented here as medians (IQR). If the measurement data met the requirements of normality and homogeneity of variance, two independent sample T tests were performed; if not, a Kruskal-Wallis H test was performed. A chi-squared (*χ*^2^) test or Fisher's exact test was used to analyze categorical data. *Post-hoc* multiple comparisons were performed to determine the origins of significant differences, and the results were adjusted by using the Bonferroni method. *P*-values of <0.05 were taken to indicate statistical significance. Graphs were produced with GraphPad Prism (Version 8.4.3, GraphPad Software Inc).

## Results

### Demographic characteristics

**Figure 1:**
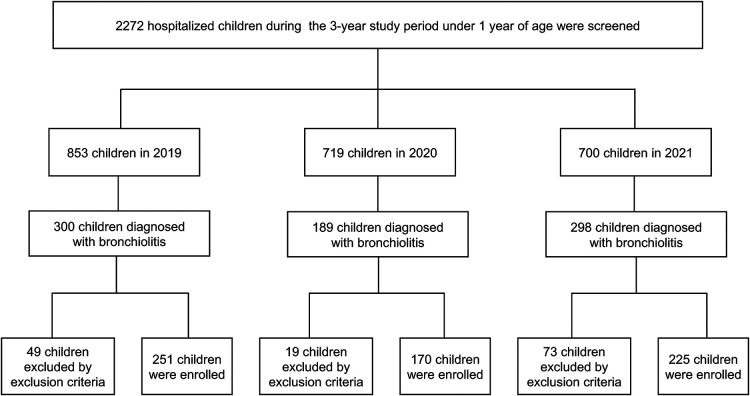
Study flow chart.

Among the 2,272 children under 1 year of age who were hospitalized during the 3-year study period, 787 (34.64%) were diagnosed with bronchiolitis: 300 (38.12%) in 2019, 189 (24.01%) in 2020, and 298 (37.87%) in 2021. Of these 787 children, 646 (82.08%) patients were included as study participants, and the other patients (17.92%) were excluded because they met the exclusion criteria (Figure 1). In 2019, 251 children were enrolled; 160 (63.75%) were male, and 167 (66.53%) were aged 1 to <6 months, 84 (33.47%) were aged 6 to <12 months. In 2020, 170 children were enrolled; 68.24% (116/170) were male, and 99 (58.24%) were aged 1 to <6 months, 71 (41.76%) were aged 6 to <12 months. In 2021, 225 children were enrolled; 160 (71.11%) were male, and 140 (62.22%) were aged 1 to <6 months, 85 (37.78%) were aged 6 to <12 months.

### Etiology

#### Predominant pathogens in the 3-year study

**Figure 2:**
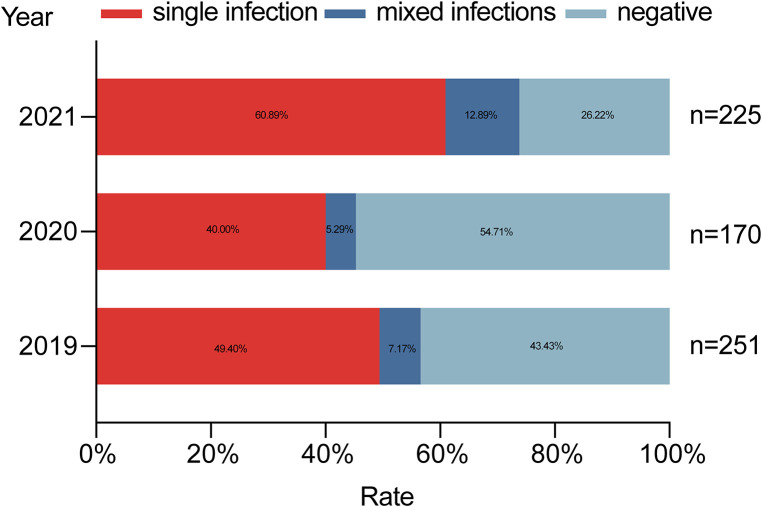
Pathogen-detection rates over the 3-year study.

In 2019, the detection rate of at least one pathogen was 56.57% (142/251); a single pathogen was detected in 49.40% (124/251) of the cases, and mixed infections were observed in 7.17% (18/251) of the cases. In 2020, the pathogen detection rate was 45.29% (77/170); a single pathogen found in 40% (68/170) of the cases, and multiple pathogens were detected in 5.29% (9/170) of the cases. In 2021, the positive detection rate was 73.78% (166/225); a single pathogen was observed in 60.89% (137/225) of the cases, and mixed pathogens were found in 12.89% (29/225) (Figure 2). The pathogen detection rate in 2021 was higher than other groups, and the difference between these rates was statistically significant (*χ*^2^ = 34.187, *p *< 0.001).

In 2019, the most common pathogen was RSV, with a positive detection rate of 33.47% (84/251). This was followed by MP at 11.95% (30/251), HBoV at 7.57% (19/251), HRV at 4.38% (11/251), PIV III at 3.59% (9/251), and hMPV at 2.39% (6/251). RSV was also the most common pathogen in 2020, with a positive rate of 15.29% (26/170). The second most common pathogen was HRV at 12.94% (22/170), followed by HBoV at 7.65% (13/170), MP at 5.29% (9/170), PIV III at 4.12% (7/170), and hMPV at 3.53% (6/170). In 2021, RSV was again the most common pathogen, with 110 (48.89%) children infected. This was followed by HRV at 16.44% (37/225), PIV III at 5.33% (12/225), hMPV at 5.33% (12/225), HBoV at 3.56% (8/225), and MP at 1.78% (4/225) (Table 1).

**Table 1:** Predominant pathogens over the 3-year study.

	RSV^a,b,c^	MP^c^	HRV^a,c^	hMPV^d^	HBoV^d^	PIV III^d^
2019	84 (33.47)	30 (11.95)	11 (4.38)	6 (2.39)	19 (7.57)	9 (3.59)
2020	26 (15.29)	9 (5.29)	22 (12.94)	6 (3.53)	13 (7.65)	7 (4.12)
2021	110 (48.89)	4 (1.78)	37 (16.44)	12 (5.33)	8 (3.56)	12 (5.33)
* χ* ^2^	48.728	20.456	18.924	2.895	4.132	0.900
*P* value	0.000	0.000	0.000	0.235	0.127	0.638

Data are presented as *n* (%). RSV, respiratory syncytial virus; MP, Mycoplasma pneumoniae; HRV, human rhinovirus; hMPV, human metapneumovirus; HBoV, human bocavirus; ADV, adenovirus; and PIV Ⅲ, parainfluenza 3.

^a^ Significant difference was observed in the pathogen-detection rate among children in 2019 and 2020.

^b^ Significant difference was observed in the pathogen-detection rate among children in 2020 and 2021.

^c^ Significant difference was observed in the pathogen-detection rate among children in 2019 and 2021.

^d^ No significant difference was observed in the pathogen-detection rate during the 3-year study period.

The positive detection rate of RSV was lowest in 2020 and highest in 2021. The rate of MP detection in 2021 was less than that in 2019 but similar to that in 2020. The rate of HRV detection in 2021 was more than that in 2019 but similar to that in 2020. There were no statistical differences in the positive rates over the 3-year study period for hMPV, HBoV, or PIV III.

#### Patterns of RSV-bronchiolitis over the 3-year study

Because RSV was the most common pathogen in each of the 3 study years, and the detection rate of this virus was markedly different between years, we further compared the positive detection rate of RSV among different age groups as well as the monthly distribution of RSV-positive cases among all age groups and in each age group among the 3 years.

**Figure 3:**
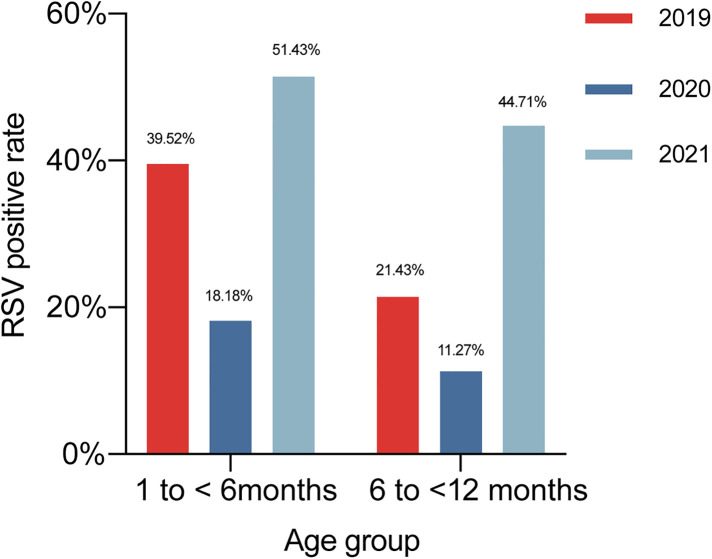
The positive RSV detection rates over the 3-year study of different age groups.

In 2019, the positive detection rate of RSV was 39.52% (66/167) in children aged 1 to <6 months, 21.43% (18/84) in those aged 6 to <12 months. In 2020, the positive detection rate of RSV was 18.18% (18/99) in children aged 1 to <6 months, 11.27% (8/71) in those aged 6 to <12 months. In 2021, the positive detection rate of RSV was 51.43% (72/140) in children aged 1 to <6 months, 44.71% (38/85) in those aged 6 to <12 months (Figure 3). In children aged 1 to <6 months, the RSV-positive rate in 2020 was lower compared with those in 2019 and 2021 (*χ*^2 ^= 27.237, *p* < 0.001). RSV-positive rate in children aged 6 to <12 months rose in 2021 compared to the previous 2 years (*χ*^2^ = 23.932, *p* < 0.001).

Regarding the monthly distribution over the 3-year study of RSV-positive cases for different age groups, we found that in 2021, the number of positive cases of RSV in all age groups as well as in each age group were increased significantly during the winter, and this increase started as early as October.

**Figure 4:**
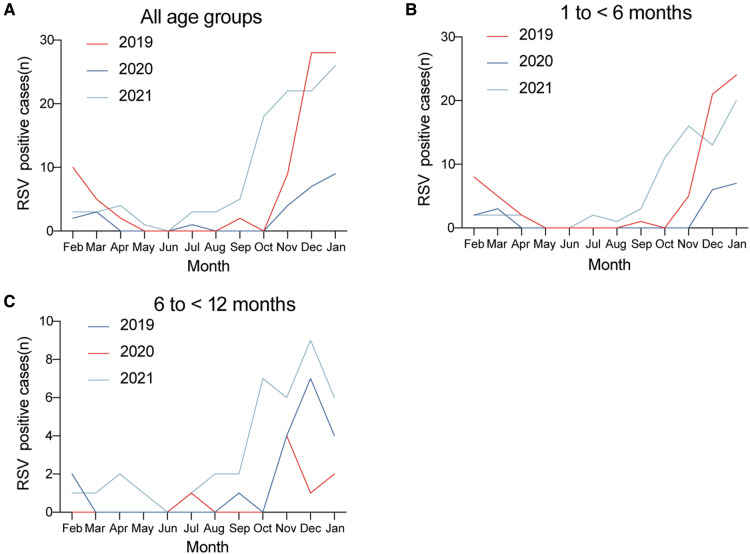
Monthly distribution of RSV-positive cases in (**A**) all age groups, (**B**) 1 to <6 months, and (**C**) 6 to <12 months over the 3-year study period.

**Figure 5:**
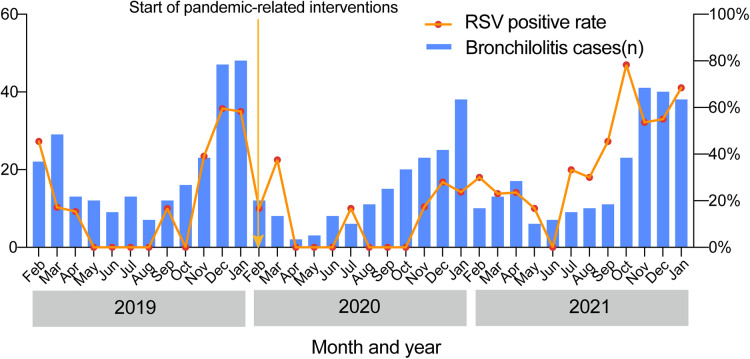
Seasonal distributions of bronchiolitis cases and the positive RSV detection rate over the 3-year study.

Finally, we compared the monthly distributions of bronchiolitis cases and the RSV-positive rate over the 3 study years (Figure 5). Longitudinally, the epidemic seasons for bronchiolitis and RSV peaked in the winter months. We found that the number of bronchiolitis cases and the positive detection rate of RSV both dropped sharply beginning in February 2020 when the lockdown measures were initiated. This was followed by increases in the winter of 2021.

#### Comparisons of the demographic and clinical characteristics of the patients enrolled in the 3-year study

The demographic and clinical characteristics of patients with bronchiolitis are shown in Table 2. There were no significant differences in patient gender among the study years. The median of age, pre-admission symptom duration and length of stay were neither shortened nor extended over the study period. Regarding the clinical manifestations, the proportion of children presenting with stuffy nose rhinorrhea in 2021 was greater than that in 2019 and 2020, while the proportion of children with gastrointestinal symptoms in 2021 was smaller than that in 2019 but similar to that in 2020. The proportions of children presenting with fever, dyspnea, tachypnea, and cyanosis were similar between years. Moreover, there were no significant differences among the years in the patient laboratory findings, including the white blood cell count, neutrophil percentage, and CRP level.

**Table 2:** Demographic and clinical characteristics of the patients in all age groups over the 3-year study.

Clinical features	2019	2020	2021	*P* value
No. of patients	251	170	225	-
**General features**				
Gender (male/female)	160/91	116/54	160/65	0.224
Age (months) (IQR)	4.20 (2.33–6.90)	5.01 (2.81–8.10)	5.07 (3.00–7.40)	0.060
Symptom duration prior to admission (day) (IQR)	6.00 (4.00–10.00)	6.00 (4.00–10.00)	5.00 (4.00–9.00)	0.458
Length of stay (day) (IQR)	7.00 (6.00–8.00)	7.00 (6.00–8.00)	7.00 (6.00–9.00)	0.125
**Clinic presentation**				
Fever [*n* (%)]^c^	77 (30.68)	59 (34.71)	65 (28.89)	0.462
Stuffy nose rhinorrhea [*n* (%)]^a,b^	122 (48.61)	97 (57.06)	165 (73.33)	0.000
Dyspnea [*n* (%)]^c^	17 (6.77)	6 (3.53)	6 (2.67)	0.076
Gastrointestinal symptoms [*n* (%)]^b^	64 (25.50)	29 (17.06)	26 (11.56)	0.000
Tachypnoea [*n* (%)]^c^	42 (16.73)	25 (14.71)	21 (9.33)	0.056
Cyanosis [*n* (%)]^c^	4 (1.59)	3 (1.76)	0 (0.00)	0.100
**Laboratory tests**				
WBC count (*10^9^/L) (IQR)	9.79 (7.45–13.21)	9.77 (7.92–11.92)	9.66 (6.66–13.03)	0.500
Percentage of neutrophils (IQR)	25.20 (16.70–39.20)	27.75 (18.70–42.83)	27.45 (18.10–39.25)	0.415
CRP count >8 mg/L [*n* (%)]	37 (14.74)	27 (15.88)	38 (16.89)	0.813

^a^ Significant difference was observed in the clinical characteristic among children in 2020 and 2021.

^b^ Significant difference was observed in the clinical characteristic among children in 2019 and 2021.

^c^ No significant difference was observed in the clinical characteristic during the 3-year study period.

We also compared the demographic and clinical characteristics among study years within each of the different age groups (Supplementary Tables S1, S2). The proportion of 1 to <6 months children presenting with stuffy nose rhinorrhea in 2021 was greater than those in the previous 2 years. The proportion of 6 to <12 months children presenting with stuffy nose rhinorrhea in 2021 was greater than that in 2019 but similar to that in 2020, whereas the proportion of such children with gastrointestinal symptoms in 2021 was smaller than that in 2019 but similar to that in 2020.Consistent with the observations for the entire study population, there were no significant differences in laboratory test results.

## Discussion

COVID-19, a coronavirus disease caused by SARS-CoV-2, broke out in Wuhan, China in December 2019 (8). SARS-CoV-2 is a highly contagious respiratory virus that spreads from person to person through contact with respiratory droplets and aerosols from infected individuals (11). In late January 2020, the Chinese government issued a national public health intervention policy, which included closing schools and other public places, prohibiting people from gathering, and encouraging mask wearing, hand washing, and disinfecting, to help prevent the spread of the epidemic. These measures also effectively suppressed the transmission of pathogens that cause bronchiolitis because the transmission pattern of bronchiolitis-causing pathogens, in particular RSV, is similar to that of SARS-CoV-2 (12). However, with the resumption of work and school, the epidemic prevention and control measures have been gradually relaxed.

In this study, we conducted a retrospective investigation of children hospitalized with bronchiolitis over 3 consecutive years (2019, 2020, and 2021) from February 1 to January 31, to compare the clinical features and etiological composition of this population before and during the COVID-19 pandemic. We found that the pathogen detection rate was the lowest in 2020, likely because 2020 was the first year of the COVID-19 pandemic and the associated interventions also reduced the spread of other respiratory pathogens. However, the pathogen detection rate in 2021 was the highest among the 3 study years, possibly because the COVID-19 control measures were eased in the second year of the COVID-19 pandemic.

In this 3-year study, we found that RSV was the predominant pathogen, a finding which is in agreement with many previous studies. Recently, a systematic review and meta-analysis of the prevalence of common respiratory viruses in children under 2 years of age with bronchiolitis in the pre-COVID-19 pandemic era found that RSV was the dominant etiologic agent of bronchiolitis (13). Correspondingly, the RSV detection rate changed in parallel with the total detection rate over our study years. In the first year of the COVID-19 pandemic, the RSV detection rate was the lowest, which is consistent with findings from previous work (14, 15). In the most severe months of the COVID-19 pandemic in 2020, there was a high uptake of enhanced hygiene and physical distancing measures. Handwashing can damage the lipid envelope that surrounds RSV, thereby impairing its ability to infect host cells (16). Another potential explanation for this difference is that there may have been a change in the ecology of respiratory viruses during the beginning of the COVID-19 pandemic. It is thought that rhinoviruses can stimulate the antiviral defenses of the mucous membrane of the respiratory tract, interfering with and blocking infection by other viruses (17). However, in the second year of the COVID-19 pandemic, the RSV detection rate increased significantly. Seasonal distribution studies conducted on participants in different age groups also found a significant increase in RSV-positive cases during the winter of 2021, and this increase began as early as October. In 2020, researchers from Princeton University and the National Institutes of Health predicted that a decline in common respiratory pathogens, such as RSV and influenza, could increase the population's susceptibility to these diseases, leading to future pandemics when they flare up again. They also predicted that RSV infections would be delayed after the end of the non-pharmaceutical interventions (NPIs) phase of the COVID-19 pandemic response, with a peak of RSV cases expected in many places during the winter of 2021–2022 (18). Our results are in line with their expectations, but this pattern is not unique to China; other regions, including Australia and Tokyo, had already observed an RSV resurgence in September 2020 and July 2021, respectively (19, 20). The timing of RSV prevalence is inconsistent in different regions, which may be related to regional and climate differences. Similarly, we also found that the number of bronchiolitis cases and the positive RSV detection rate dropped sharply beginning in February 2020 when the lockdown measures were taken and then increased again when the intervening measures were relaxed, especially in the winter of 2021. The resurgence of RSV may be attributed to “immune debt”, a term established to characterize the lack of protective immunity resulting from prolonged periods of low exposure to a certain pathogen, making a larger percentage of the population susceptible to the disease (21). Due to the transitory immunity established by RSV through virus exposure, maternal antibodies deplete quickly, and without seasonal exposure, immunity declines and susceptibility to subsequent and potentially more severe infections rises (22).

The positive rate of MP detection in 2021 was lower than that in 2019 but similar to that in 2020. This result is similar to those from a study conducted in Chengdu, China. In that study, researchers analyzed the prevalence of MP infection in children with respiratory symptoms from January 2017 to December 2020, and they found that public health measures adopted during the COVID-19 pandemic response could effectively control the spread of MP (23). MP, which is the smallest independent pathogenic microorganism between bacteria and viruses, often affects school-aged children. It continues to circulate and spread periodically, exhibiting an epidemic peak every 3 to 7 years.MP is a common causative pathogen of children's community-acquired pneumonia and, like RSV, is an important causative pathogen of bronchiolitis. However, in our study, the prevalence of MP was not found to rebound like that of RSV. It is unclear whether this occurrence is because of the epidemiological characteristics of MP itself or a low susceptibility to MP in children under 1 year of age.

Our study found that the detectable rate of HRV in 2021 was higher than that in 2019 but similar to that in 2020. Rhinoviruses are non-enveloped organisms that remain on environmental surfaces longer than many other pathogens (24), and they are not sensitive to ethanol-based disinfectants (25). So it is possible that these factors contributed to the rise in the positive rate of rhinovirus, despite the widespread use of disinfectants during the pandemic. However, the precise mechanism is still unknown.

We found that, except for differences in the percentage of patients with stuffy nose rhinorrhea, and gastrointestinal symptoms, there were no significant differences in the prevalence of bronchiolitis clinical features over the 3-year study period. The increase in the percentage of patients with bronchiolitis who reported stuffy nose rhinorrhea during the COVID-19 pandemic from that in 2019 may be due to the increased detection rate of rhinovirus, given that the most common symptoms of HRV infection are runny nose, nasal congestion, sore throat, and cough. During the COVID-19 pandemic, the percentage of patients with bronchiolitis who reported gastrointestinal symptoms (vomiting/diarrhea) decreased, possibly owing to the small sample size and the COVID-19 intervention measures.

Our study has some limitations. First, we included only patients with bronchiolitis who had undergone nasopharyngeal secretion examinations, and we did not enroll any patients with only a bacterial infection. Second, our samples were all collected from Suzhou City; thus, they do not fully reflect the epidemiological changes owing to geographical limitations. Finally, all of the children enrolled in our study were inpatients because it was not possible to obtain the epidemiology of respiratory pathogens from outpatients.

## Conclusion

After the implementation of interventions to tackle the spread of SARS-CoV-2 infection, we observed a significant decline in the pathogen detection rate and RSV-positive rate in the first year of the COVID-19 pandemic. A rebound appeared to take place in the second year of the COVID-19 pandemic. The detection rate of MP decreased while the positive rate of HRV increased during COVID-19 pandemic as compared with those rates before the pandemic. The difference in pathogen composition also led to an observable difference in the clinical characteristics of children with bronchiolitis before and during the epidemic. At present, the global COVID-19 pandemic remains serious. Given that the impact of the relaxation of preventative measures against COVID-19 on the diseases caused by other respiratory viruses, including RSV, in the susceptible population is unknown (26–28), it is of great importance to strengthen disease prevention, control, and monitoring systems.

## Data Availability

The original contributions presented in the study are included in the article/**Supplementary Material**, further inquiries can be directed to the corresponding author/s.
