# Supplementary material for: Clinical characteristics and etiology of children with bronchiolitis before and during the COVID-19 pandemic in Suzhou, China
Source: Front Pediatr. 2022 Nov 14;10:974769. doi: 10.3389/fped.2022.974769 (PMC9703066; doi:10.3389/fped.2022.974769)
Supplement: Supplementary file 1 [file Table1.pdf]

**Supplementary Table 1. Demographic and clinical characteristics of the patients aged 1 to < 6 months enrolled in the 3-year study**

| Clinical features | 2019 | 2020 | 2021 | *P* value |
|---|---|---|---|---|
| No. of patients | 167 | 99 | 140 | - |
| General features | | | | |
| Gender(male/female) | 104/63 | 77/22 | 98/42 | 0.029 |
| Age(months)(IQR) | 3.00(1.87~4.20) | 3.08(1.90~4.43) | 3.77(2.40~4.86) | 0.013 |
| Symptom duration prior to admission(day)(IQR) | 5.00(4.00~9.00) | 6.00(4.00~10.00) | 5.00(4.00~8.00) | 0.242 |
| Length of stay(day)(IQR) | 7.00(6.00~8.00) | 7.00(6.00~8.00) | 7.00(6.00~8.00) | 0.468 |
| Clinic presentation | | | | |
| Fever [n (%)] [c] | 41(24.55) | 25(25.25) | 23(16.43) | 0.151 |
| Stuffy nose rhinorrhea [n (%)] [ab] | 82(49.10) | 51(51.52) | 106(75.71) | 0.000 |
| Dyspnea [n (%)] [c] | 11(6.59) | 5(5.05) | 4(2.86) | 0.344 |
| Gastrointestinal symptoms [n (%)] [c] | 40(23.95) | 18(18.18) | 19(13.57) | 0.067 |
| Tachypnoea [n (%)] [c] | 26(15.57) | 14(14.14) | 12(8.57) | 0.170 |
| Cyanosis [n (%)] [c] | 3(1.80) | 3(3.03) | 0(0.00) | 0.119 |
| Laboratory tests | | | | |
| WBC count(*$10^9$/L) (IQR) | 9.80(7.27～12.90) | 9.64(8.03～11.70) | 9.58(6.63～13.17) | 0.670 |
| Percentage of neutrophils (IQR) | 21.30(15.50～34.90) | 24.70(15.20～39.50) | 25.30(15.50～35.60) | 0.433 |
| CRP count >8mg/L [n (%)] | 20(11.98) | 11(11.11) | 21(15.00) | 0.618 |

[a] Significant difference was observed in the clinical characteristic among children in 2020 and 2021.
[b] Significant difference was observed in the clinical characteristic among children in 2019 and 2021.
[c] No significant difference was observed in the clinical characteristic during the 3-year study period.
