# Supplementary material for: Clinical characteristics and etiology of children with bronchiolitis before and during the COVID-19 pandemic in Suzhou, China
Source: Front Pediatr. 2022 Nov 14;10:974769. doi: 10.3389/fped.2022.974769 (PMC9703066; doi:10.3389/fped.2022.974769)
Supplement: Supplementary file 2 [file Table2.pdf]

Supplementary Table 2. Demographic and clinical characteristics of the patients aged 6 to < 12 months enrolled in the 3-year study

Clinical features	2019	2020	2021	*P* value
No. of patients	84	71	85	-
General features				
Gender(male/female)	56/28	39/32	62/23	0.476
Age(months)(IQR)	8.14(6.90~10.57)	8.83(7.07~10.13)	8.30(6.85~10.67)	0.745
Symptom duration prior to admission(day)(IQR)	7.00(4.00~15.00)	6.00(4.00~8.00)	6.00(4.00~10.00)	0.337
Length of stay(day)(IQR)	7.00(6.00~9.00)	7.00(6.00~8.00)	7.00(7.00~9.00)	0.073
Clinic presentation				
Fever [n (%)] [b]	36(42.86)	34(47.89)	42(49.41)	0.674
Stuffy nose rhinorrhea [n (%)] [a]	40(47.62)	46(64.79)	59(69.41)	0.010
Dyspnea [n (%)] [b]	6(7.14)	1(1.41)	2(2.35)	0.146
Gastrointestinal symptoms [n (%)] [a]	24(28.57)	11(15.49)	7(8.24)	0.002
Tachypnoea [n (%)] [b]	16(19.05)	11(15.49)	9(10.59)	0.303
Cyanosis [n (%)] [b]	1(1.19)	0(0.00)	0(0.00)	0.646
Laboratory tests				
WBC count(*10^9/L) (IQR)	9.74(7.77~13.70)	10.23(7.61~12.08)	9.65(7.06~12.35)	0.711
Percentage of neutrophils (IQR)	34.35(22.83~55.55)	32.10(22.40~46.10)	31.40(22.70~45.20)	0.311
CRP count >8mg/L [n (%)]	17(20.24)	16(22.54)	17(20.00)	0.915

[a] Significant difference was observed in the clinical characteristic among children in 2019 and 2021.

[b] No significant difference was observed in the clinical characteristic during the 3-year study period.
